# Milademetan is a highly potent MDM2 inhibitor in Merkel cell carcinoma

**DOI:** 10.1172/jci.insight.160513

**Published:** 2022-07-08

**Authors:** Varsha Ananthapadmanabhan, Thomas C. Frost, Kara M. Soroko, Aine Knott, Brianna J. Magliozzi, Prafulla C. Gokhale, Vijaya G. Tirunagaru, Robert C. Doebele, James A. DeCaprio

**Affiliations:** 1 Department of Medical Oncology, Dana-Farber Cancer Institute, Boston, Massachusetts, USA.; 2 Department of Medicine, Brigham and Women’s Hospital and Harvard Medical School, Boston, Massachusetts, USA.; 3 Program in Virology, Graduate School of Arts and Sciences, Harvard University, Cambridge, Massachusetts, USA.; 4 Experimental Therapeutics Core at Dana-Farber Cancer Institute, Boston, Massachusetts, USA.; 5 Rain Therapeutics, Newark, California, USA.

**Keywords:** Oncology, Therapeutics, Apoptosis, Skin cancer, p53

## Abstract

Merkel cell carcinoma (MCC) is an aggressive neuroendocrine carcinoma of the skin with 2 etiologies. Merkel cell polyomavirus (MCPyV) integration is present in about 80% of all MCC. Virus-positive MCC (MCCP) tumors have few somatic mutations and usually express WT p53 (*TP53*). By contrast, virus-negative MCC (MCCN) tumors present with a high tumor mutational burden and predominantly UV mutational signature. MCCN tumors typically contain mutated *TP53*. MCCP tumors express 2 viral proteins: MCPyV small T antigen and a truncated form of large T antigen. MCPyV ST specifically activates expression of MDM2, an E3 ubiquitin ligase of p53, to inhibit p53-mediated tumor suppression. In this study, we assessed the efficacy of milademetan, a potent, selective, and orally available MDM2 inhibitor in several MCC models. Milademetan reduced cell viability of WT p53 MCC cell lines and triggered a rapid and sustained p53 response. Milademetan showed a dose-dependent inhibition of tumor growth in MKL-1 xenograft and patient-derived xenograft models. Here, along with preclinical data for the efficacy of milademetan in WT p53 MCC tumors, we report several in vitro and in vivo models useful for future MCC studies.

## Graphical abstract

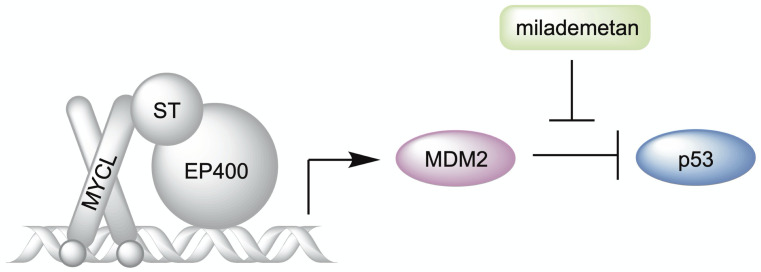

## Introduction

The tumor suppressor protein p53 plays an essential role in the cancer cell cycle (1, 2). Approximately 50% of all cancers have a mutation in the *TP53* gene (2, 3). In cells with WT p53, activation of p53 in response to cellular stress or DNA damage leads to transactivation of many p53 target genes, resulting in cell cycle arrest, apoptosis, or senescence (1, 2, 4). Levels of WT p53 in the cell are regulated by a negative feedback loop. Activated p53 binds to the p53 response elements in the *MDM2* gene, leading to an increase in *MDM2* expression. The MDM2 protein, an E3 ubiquitin ligase, in turn binds and ubiquitinates p53, leading to its degradation by the proteasome (5–9). Therefore, MDM2 is an important regulator of p53 and can be an effective therapeutic target in cancers with WT p53. Pharmacological inhibition of MDM2 for stabilization of p53 has been of interest for several years, particularly for cancers with MDM2 amplification, including liposarcoma, Ewing’s sarcoma, osteosarcoma, and leukemia (2, 10–12). Several MDM2 inhibitors targeting the MDM2-p53 interaction are currently in clinical trials for treatment of these cancers (2), although none have received FDA approval for any therapeutic use.

Merkel cell carcinoma (MCC) is a highly aggressive neuroendocrine carcinoma of the skin with a high morbidity rate (13–15). MCC frequently metastasizes to lymph nodes and distant organs, including liver, bone, pancreas, lung, and brain (13–15). MCC has 2 distinct etiologies. Clonally integrated Merkel cell polyomavirus (MCPyV) is present in virus-positive MCC (MCCP). These tumors have a low tumor mutational burden, with nearly normal diploid genomes (14–20). In contrast, virus-negative MCC (MCCN) tumors are caused by chronic UV light exposure, leading to a high mutational burden with a strong UV mutational signature (14–20). Despite these distinct etiologies, both forms of MCC exhibit similar histology, aggressive phenotype, and response to therapy, suggesting that they perturb similar oncogenic pathways. While MCCN typically contain loss-of-function mutations in *TP53* and the retinoblastoma tumor suppressor (*RB1*), MCCP usually contains WT p53 and retinoblastoma (RB) proteins (14, 15, 20–22). About 80% of MCC tumors are MCCP, most of which have WT p53 (16, 18, 20, 23–26).

MCCP tumors express 2 viral proteins: small T antigen (ST) and a truncated form of the large T antigen (LT) (14, 15, 20). Specific binding of MCPyV LT to RB leads to inactivation of RB protein and activation of E2F target genes that contribute to entry and progression into the cell cycle (14, 15, 20–22, 27). Moreover, through its association with RB protein, MCPyV LT activates p53 (14, 20, 28). To counteract p53 activation, MCPyV ST recruits MYCL, a MYC paralog, to the EP400 histone acetyltransferase and chromatin remodeling complex to transactivate many downstream target genes (14, 20, 29). One such ST-MYCL-EP400 target gene is MDM2, the negative regulator of p53 (8, 9, 20, 28, 29). Functional inactivation of human p53 protein in MCCP tumors or murine *Trp53* deletion in MCCP mouse models is required to generate a MCC phenotype (30). Thus, restoring p53 function in MCCP tumors could be a beneficial antitumor strategy.

Current treatment options for MCC include surgery and radiation therapy for localized tumors (14). Metastatic MCC is highly responsive to chemotherapy in the initial stages; however, progression-free survival after chemotherapy is limited (31). Checkpoint blockade therapy has been effective for advanced disease, but limited responsiveness is an issue (32). Therefore, there is a need for the identification and development of targeted therapeutic agents that could be effective in MCC.

In MCCP cell lines and xenograft tumors with WT p53, inhibition of MDM2 and its paralog MDM4 has been found to be an effective antitumor approach (14, 28). However, there are no MDM2 or MDM4 inhibitors that have received FDA approval for treatment of MCC. Therefore, there is a need for the development and identification of potent inhibitors with therapeutic potential in patients with MCC. Milademetan (DS-3032, RAIN-32) is a highly potent, orally available, small-molecule inhibitor of the p53-MDM2 interaction. Prior data indicate that milademetan restored WT p53 activity in in vitro and in vivo cancer models with WT p53 (33–36). Here, we demonstrate the activity of milademetan toward reactivating WT p53 in MCCP MCC using established MCC cell lines, patient-derived cell lines (PDCLs), and multiple patient-derived xenograft (PDX) models.

## Results

### MCC cell lines with WT p53 are sensitive to MDM2 inhibition.

**Figure 1:**
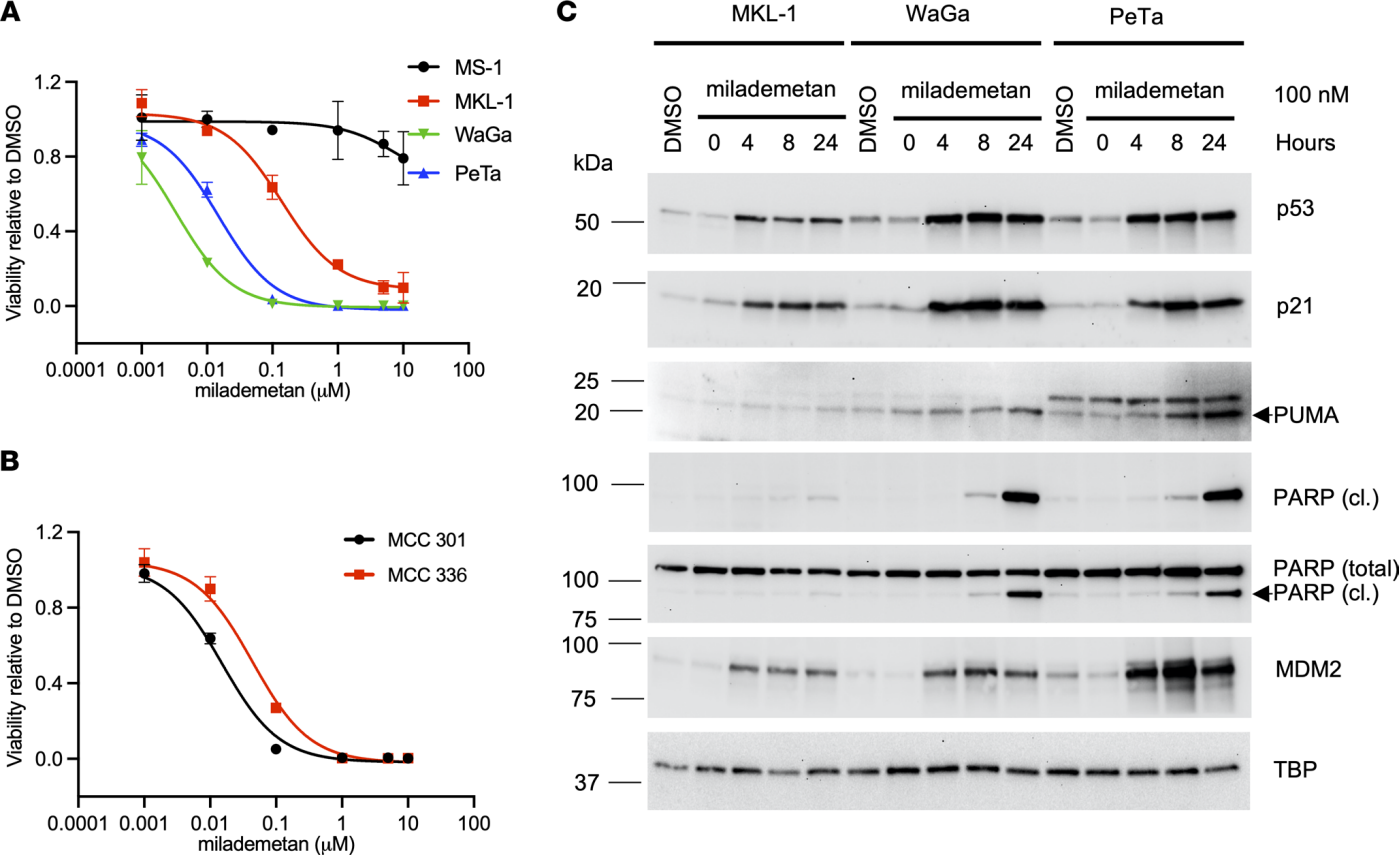
MCCP cell lines with WT p53 are sensitive to milademetan treatment. (**A**) MKL-1, WaGa, PeTa, and MS-1 cell lines and (**B**) MCC PDCLs MCC-301 and MCC 336 were treated with indicated doses of milademetan, and the Cell Titer Glo assay was performed to assess the effect on viability after 3 days of treatment. Each assay was performed in triplicate, and 3 biological replicates were performed. Data are shown as the mean ± SD. (**C**) MCC MKL-1, WaGa, and PeTa cell lines were treated with 100 nM milademetan, and p53 response was analyzed using WB analysis for the indicated proteins. TBP was used as a loading control. Representative WB of *n =* 2. PARP (cl.) indicates cleaved PARP.

To test the effect of milademetan on MCC cell viability, we treated 4 established virus-positive (MCCP) cell lines with milademetan for 3 days, followed by measurement of cell viability using a highly sensitive ATP-based viability assay. Three MCCP cell lines, MKL-1, WaGa, and PeTa, that contain WT p53 were sensitive to nanomolar concentrations of milademetan treatment, whereas the MCCP p53 mutant cell line MS-1 was highly resistant (Figure 1A and Table 1) (21). Similarly, MKL-1, WaGa, and PeTa cells but not MS-1 cells were sensitive to the MDM2 inhibitor Nutlin-3a (Supplemental Figure 1A and Supplemental Table 1; supplemental material available online with this article; https://doi.org/10.1172/jci.insight.160513DS1) (37). The drug responses for each cell line were analyzed by the half-maximal inhibitory concentration (IC_50_) and AUC values. We observed that the more sensitive the cell line, the lower the absolute IC_50_ and AUC values (Table 1 and Supplemental Table 1). Even among cell lines sensitive to MDM2 inhibition, there was a range of responses observed, with MKL-1 being the least sensitive and WaGa being the most sensitive to MDM2 inhibition (Figure 1A, Supplemental Figure 1A, Table 1, and Supplemental Table 1).

In addition to established MCC cell lines, we carried out similar viability assays for 2 MCCP PDCLs that contain WT p53. These cell lines, MCC-301 and MCC-336, were highly sensitive to milademetan and Nutlin-3a treatment (Figure 1B, Supplemental Figure 1B, Table 2, and Supplemental Table 2). The absolute IC_50_ and AUC values also denote that milademetan was highly potent, as compared with Nutlin-3a, in all the MCC cell lines with WT p53 that were tested (Tables 1 and 2 and Supplemental Tables 1 and 2).

### MDM2 inhibition by milademetan activates the p53 response in MCC cell lines with WT p53.

We analyzed the effect of milademetan on activation of the p53 response by Western blot (WB) across 3 sensitive cell lines: MKL-1, WaGa, and PeTa (Figure 1C). Levels of tumor suppressor p53 protein accumulated in all 3 cell lines within hours of treatment with 100 nM milademetan. The WaGa and PeTa cell lines showed higher levels of p53 in response to milademetan compared with the MKL-1 cell line. Interestingly, the MKL-1 cell line also had lower steady-state levels of p53 as compared with the more sensitive WaGa and PeTa lines (Figure 1C and Supplemental Figure 1C). We assessed the protein levels of genes transactivated by p53, including the cell cycle inhibitor p21 (encoded by the gene *CDKN1A*), the proapoptotic protein PUMA (encoded by the gene *BBC3*), and MDM2 (1, 4–7, 38–40). Milademetan treatment led to an increase in levels of p21 and MDM2 across all 3 cell lines. Levels of PUMA increased within 24 hours after milademetan treatment in WaGa and PeTa cell lines, but this was not observed with the least sensitive MCC cell line, MKL-1. Milademetan also led to an accumulation of cleaved PARP in WaGa and PeTa cell lines as early as 8 hours after treatment, indicating an apoptotic response. Lower levels of cleaved PARP accumulation were also observed in the MKL-1 cell line at later time points, 24 hours after milademetan treatment (Figure 1C). The level of the p53 response across the 3 cell lines correlated with their degree of sensitivity to milademetan treatment (Figure 1A and Table 1). These data collectively suggest that MDM2 inhibition by milademetan in MCC cell lines with WT p53 activates p53 and leads to an apoptotic response.

### Milademetan activity in MCC requires the presence of WT and functional p53.

**Figure 2:**
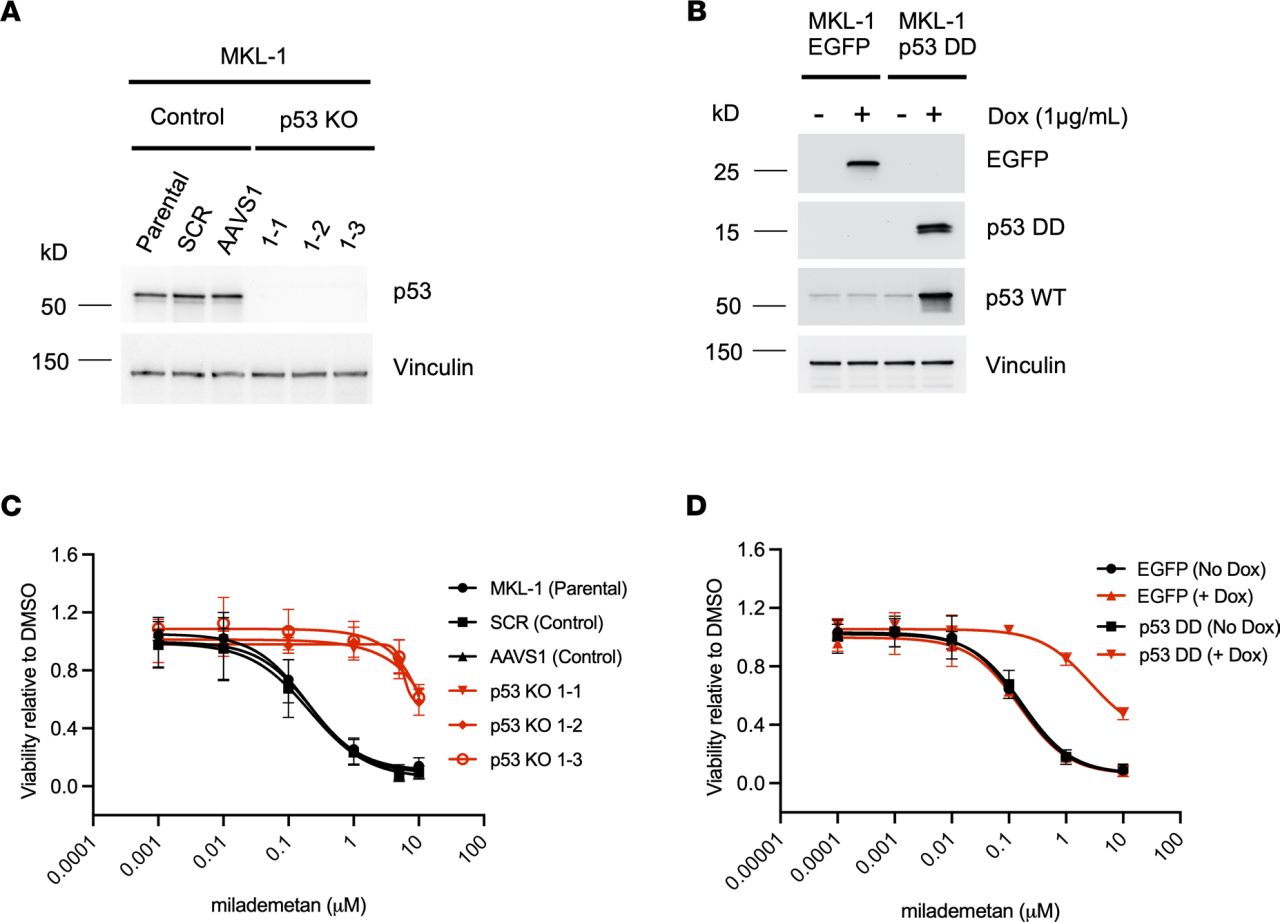
MCC cell lines devoid of p53 or expressing dominant negative p53 are resistant to milademetan treatment. (**A**) WB indicating levels of p53 in MKL-1 control or p53-KO cell lines. Vinculin was used as a loading control. (**B**) WB indicating levels of EGFP, WT p53, and dominant-negative p53 (p53 DD) with or without induction with doxycycline (dox). Vinculin was used as a loading control. (**C** and **D**) Cell lines were treated with milademetan followed by analysis of cell viability after 3 days of milademetan treatment. Each assay was performed in triplicate and 3 biological replicates were performed. Data are shown as the mean ± SD.

To test the requirement of WT p53 for sensitivity to milademetan, we generated MKL-1 p53–KO lines using CRISPR/Cas9 technology. In addition, we generated MKL-1 cell lines that express either a murine dominant-negative form of p53 (p53 DD) or EGFP under the influence of a doxycycline-inducible (dox-inducible) promoter. When expressed, the p53 DD form can bind to and inactivate the endogenous WT p53 (28, 41, 42). The levels of p53 protein across control, KO, and the p53 DD lines were analyzed by WB (Figure 2, A and B). We tested these cell lines in viability assays using milademetan and Nutlin-3a. The MKL-1 p53–KO lines and the dox-inducible cell line expressing p53 DD were resistant to milademetan and Nutlin-3a, demonstrating the requirement for intact p53 (Figure 2, C and D, and Supplemental Figure 2, A and B). Moreover, the nontargeting scramble (SCR) control, AAVS1 control, or dox-inducible EGFP-expressing control cell lines had similar IC_50_ and AUC values compared with parental MKL-1 cells (Tables 1, 3, and 4 and Supplemental Tables 3 and 4).

### Antitumor activity of milademetan in an MKL-1 xenograft model.

**Figure 3:**
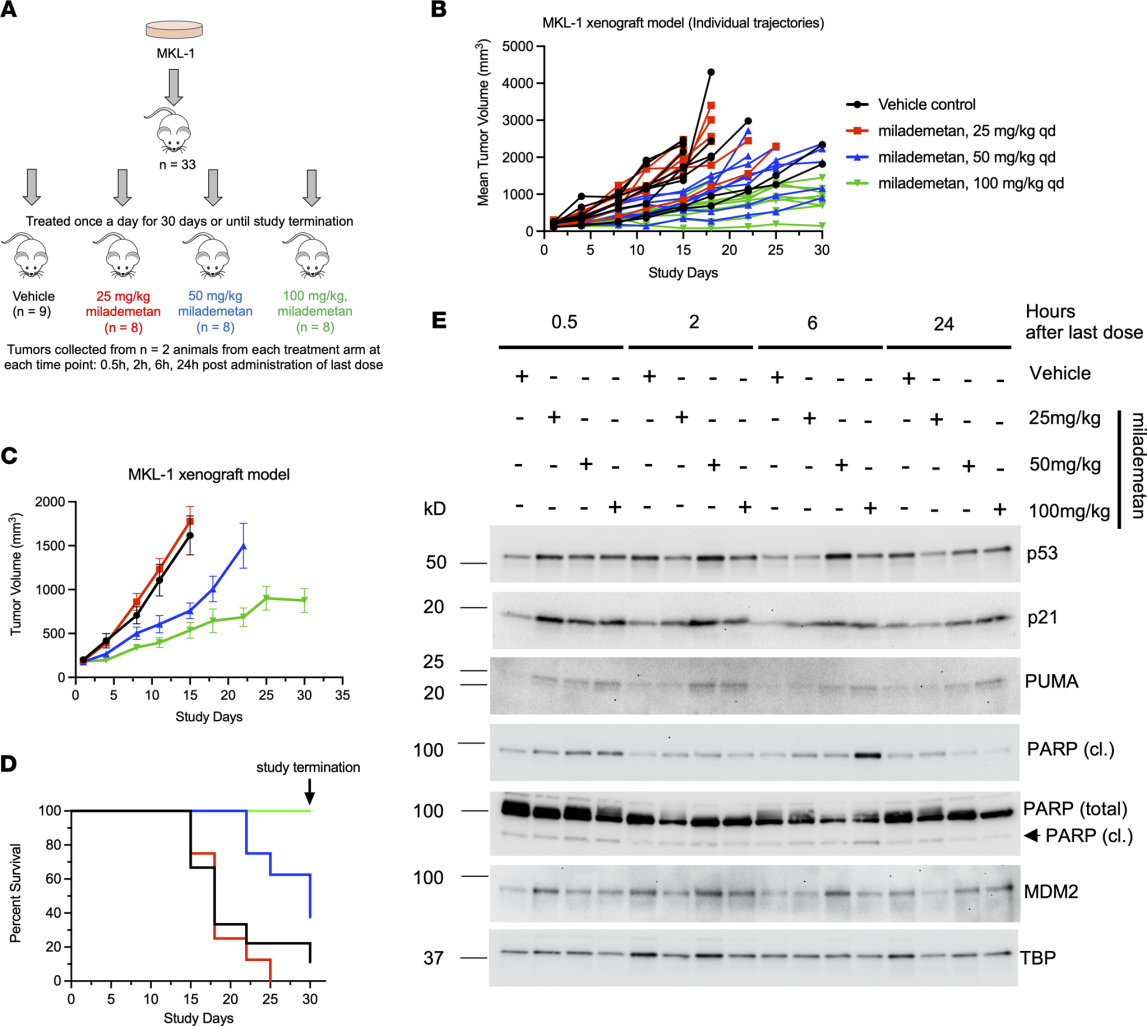
Antitumor activity of milademetan in an MKL-1 Xenograft tumor model. (**A**) Schematic representation of the study carried out in the MCC MKL-1 xenograft model treated with either vehicle or 3 different doses of milademetan. (**B**) Effect of vehicle or indicated doses of milademetan treatment on individual tumor trajectories in the MCC MKL-1 xenograft study. Data were plotted until tumor volumes reached maximal permissible size or until study termination. (**C**) Mean xenograft tumor volumes of mice treated with vehicle or indicated doses of milademetan. Data were plotted until at least 8–9 mice per treatment group were alive. Data are shown as the mean ± SEM. (**D**) Kaplan-Meier survival curves of the mice in the vehicle- or milademetan-treated groups throughout the duration of the MCC MKL-1 xenograft study. (**E**) WB analysis of 16 different tumors obtained from 16 individual mice at the indicated time points after the last dose of vehicle or milademetan was administered showing levels of indicated proteins. PARP (cl.) indicates cleaved PARP.

To assess the efficacy of milademetan in vivo, we used an MKL-1 xenograft model. Mice were either treated with vehicle or 3 different doses of milademetan, including doses of 25 mg/kg, 50 mg/kg, or 100 mg/kg once a day for 30 days (Figure 3A and Supplemental Excel file 1). The study was allowed to progress until tumors reached a maximum permissible size of 2000 mm^3^ or when the predetermined study end- point of 30 days was reached. Mice treated with the doses of 50 or 100 mg/kg showed reduction of tumor volume in this model (Figure 3, B and C; Table 5; and Supplemental Excel file 2). Kaplan-Meier survival analysis showed that mice receiving 50 or 100 mg/kg milademetan also survived for a significantly longer duration compared with that of mice that received either vehicle or 25 mg/kg milademetan (Figure 3D and Table 6). Throughout the duration of the xenograft study, we did not observe any significant changes in the weight of the mice across all treatment groups and no other adverse effects were observed (Supplemental Figure 3B and Supplemental Excel file 3).

Prior to the study endpoint, tumors were collected from 2 animals from each treatment group at 0.5, 2, 4, or 6 hours after the last dose of vehicle or milademetan was administered (Figure 3A). Lysates obtained from the tumor samples were blotted to assess the p53 response (Figure 3E and Supplemental Figure 3C). We observed an increase in levels of the p53 target gene products, including p21 and PUMA, at both early and late time points after milademetan administration across all 32 tumors analyzed, indicating an active p53 response in this model (Figure 3E and Supplemental Figure 3C). We observed modest changes in levels of MDM2, and the p53 levels remained stable with milademetan treatment. We also observed increased levels of cleaved PARP with time and increasing doses of milademetan. The peak increase in cleaved PARP was observed at 6 hours after treatment with milademetan (Figure 3E and Supplemental Figure 3C). In 1 of the 2 tumors obtained from mice treated with 50 mg/kg milademetan from the MKL-1 xenograft study, we observed an increase in cleaved PARP levels 2 hours after treatment (Supplemental Figure 3C).

### Antitumor activity of milademetan in WT p53 MCC PDX models.

**Figure 4:**
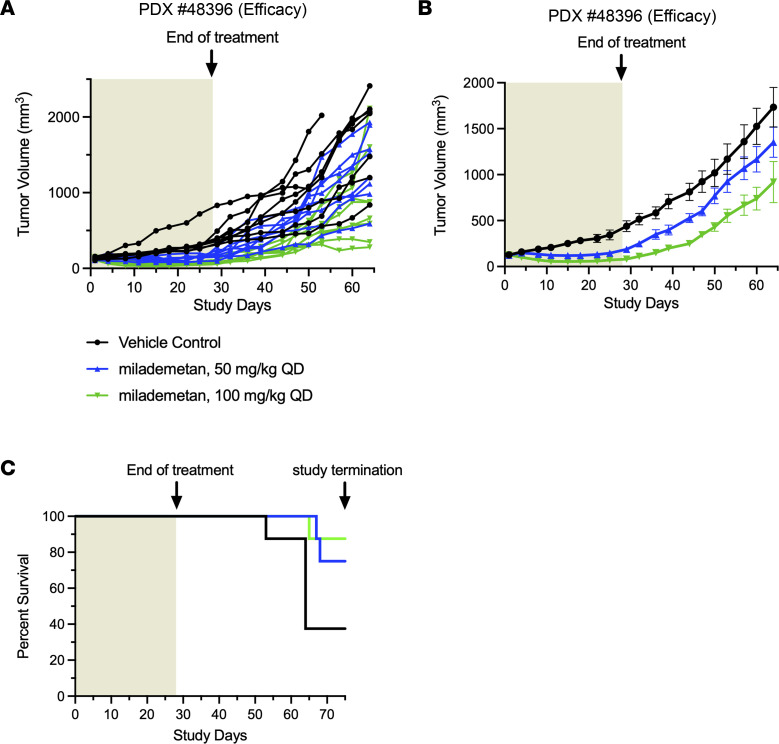
Antitumor activity of milademetan in a WT p53 PDX model. (**A**) MCC PDX 48396 individual tumor trajectories. Tumor volumes of mice treated with vehicle or indicated doses of milademetan during and after the course of treatment are shown. The shaded area indicates days when mice were treated with either vehicle or the indicated milademetan dose once a day. Data were plotted until study termination. (**B**) MCC PDX 48396 mean tumor volumes of mice treated with vehicle or indicated doses of milademetan during and after the course of treatment. Data were plotted for the period when all mice in every treatment group were alive. Data are shown as the mean ± SEM. (**C**) Kaplan-Meier survival curves of the mice in the vehicle- or milademetan-treated groups throughout the duration of the MCC PDX 48396 study.

To assess the efficacy of milademetan in vivo, we developed several MCC PDX models. Initially, we performed a pilot study using PDX models 33043, 48396, and 96712. All 3 of these PDX models contain WT p53. For this pilot study, we tested each of the 3 PDX models in a single mouse (1 × 1 study) and treated each model with either vehicle or 100 mg/kg milademetan for 21 to 28 days. Tumor volumes were measured during and following withdrawal of treatment. Milademetan was highly potent in reducing tumor volumes in all 3 MCC PDX models tested (Supplemental Figure 4A and Supplemental Excel file 4). An efficacy study was performed using the PDX 48396 model. Mice were treated for 28 days and followed after treatment withdrawal to assess for tumor regrowth. The study was terminated on day 75 or when tumors reached a maximum permissible size of 2000 mm^3^, whichever was earlier (Figure 4, A and B, and Supplemental Excel file 5). Tumor volumes of mice treated with 50 mg/kg or 100 mg/kg milademetan were significantly reduced compared with those of vehicle-treated mice at the 29-day time point (Figure 4, A and B, and Table 7). Tumor volumes remained significantly reduced, even by day 64, 35 days after treatment was withdrawn (Figure 4, A and B, and Table 8). Kaplan-Meier survival analysis showed that mice receiving 100 mg/kg milademetan survived for a significantly longer duration than mice that received vehicle doses (Figure 4C and Table 9). Throughout the duration of the PDX efficacy study, we did not observe any significant weight changes or any visible adverse effects in mice across all treatment groups (Supplemental Figure 4B and Supplemental Excel file 6).

GDF-15 (also known as MIC-1) is a secreted protein belonging to the TGF-β superfamily and a well-described known p53 target gene (28, 43, 44). GDF-15 is released from tumor cells and can be detected by analyzing serum or plasma samples (36, 44). Levels of human GDF-15 were assessed in plasma obtained from mice treated with vehicle or milademetan. We observed a dose-dependent increasing trend in GDF-15 levels 24 hours after milademetan administration on day 4 but not at 6 hours (Supplemental Figure 4C). However, the day 4, 24-hour data were not statistically significant. GDF-15 levels did not change 24 hours after milademetan administration on day 21 of dosing, likely due to reduced tumor sizes (Supplemental Figure 4D). Collectively, these data suggest that milademetan is highly potent in the in vivo MCC models.

## Discussion

Current treatment options for MCC include surgery and radiation therapy for localized tumors and checkpoint blockade therapy for advanced disease (14). Platinum-based and other forms of chemotherapy have a high rate of response in MCC; however, this response has a short duration, and tumors quickly become unresponsive (31). No targeted therapies have been shown to date to be effective in MCC clinical trials. Given that *TP53* is often WT in MCC tumors, inhibition of MDM2 with activation of a strong p53 response could be beneficial (14, 28).

Milademetan is a novel, highly potent MDM2 inhibitor, with activity in restoring WT p53 response in several WT p53 in vitro and in vivo cancer models and in a phase I clinical study (33–36). Clinical trials for milademetan efficacy in liposarcoma, acute myeloid leukemia, lymphoma, and advanced solid tumors have been completed or are in progress (NCT04979442, NCT01877382, NCT03671564). Navtemadlin (KRT-232, AMG-232) is an MDM2 inhibitor in clinical trial recruiting patients with MCC (NCT03787602).

We observed that milademetan inhibited growth of MCCP cell lines with WT p53 (Figure 1) but not the MCCP p53 mutant cell line MS-1, the MKL-1 p53–KO cell lines, or MKL-1 cell line expressing dox-inducible p53 DD (Figures 1 and 2). Different cancer cell lines with WT p53 have varying sensitivities to milademetan, with IC_50_ values ranging from 9 nM to 223 nM (33). Interestingly, MCC cell lines have much lower steady-state levels of MDM2 as compared with those of the osteosarcoma cell line SJSA-1 and the choriocarcinoma cell line JAR with MDM2 amplification (Supplemental Figure 1C), but they have a similar range of milademetan IC_50_ values. In WT p53 AML cell lines, the levels of MDM2 protein expression positively correlated with Nutlin-3a–mediated induction of apoptosis, suggesting that levels of MDM2 could be a tool to predict sensitivity in AML (45). However, MDM4 and not MDM2 protein levels varied across MCCP and MCCN cell lines (Supplemental Figure 1C). Although dual inhibition of MDM2 and MDM4 in MCCP cell lines and xenograft models was more effective than MDM2 inhibition alone (28), it needs to be determined whether MDM4 protein levels can affect sensitivity to MDM2 inhibition in MCCP cell lines. Additionally, the MCCP MKL-1 cell line has lower levels of p53 as compared with the more sensitive WaGa and PeTa cell lines (Figure 1C and Supplemental Figure 1C). Therefore, the reason for varying sensitivities of the different MCCP lines tested in this study to MDM2 inhibitor treatment may reflect differential levels of p53, but this needs further analysis. WBs revealed that milademetan was highly potent in MCC and triggered a p53-dependent apoptotic response. Although the highly sensitive WaGa and PeTa cell lines showed a robust activation of the p53 response and an accumulation of apoptotic markers PUMA and cleaved PARP, treatment with milademetan for a longer duration or with a higher concentration may be necessary to induce a similar effect in the MKL-1 cell line, which is the least sensitive to milademetan treatment (Figure 1, A and C and Table 1).

The PDCL MCC-301, which is highly sensitive to MDM2 inhibition (Figure 1B, Supplemental Figure 1B, Table 2, and Supplemental Table 2), was derived from PDX 48396. This cell line will be particularly useful for predicting the effects of other targeted inhibitors in the MCC PDX 48396 model.

In the PDX study, we observed an increasing trend of human GDF-15 levels 24 hours after administration of milademetan on day 4 but not on day 21 (Supplemental Figure 4, C and D). This could be because by day 21 the tumor size in milademetan-treated animals was already reduced, leading to decreased levels of secreted human GDF-15. We were also unable to see any changes in human GDF-15 6 hours after administration of milademetan on day 4 (Supplemental Figure 4C). Although pharmacodynamic markers in the tumor could possibly be detected at earlier time points, circulating tumor proteins probably require additional time for detection. In concert with this, in normal healthy adults given a dose of the MDM2 inhibitor navtemadlin, peak GDF-15 levels were observed after a lag time of 8–12 hours after administration (46). Further studies focused on understanding the kinetics of GDF-15 after administration of MDM2 inhibitors are essential for determining if GDF-15 could be an important clinical indicator for tracking an active p53 response in MCC.

Treatment with MDM2 inhibitors can lead to stabilization of the p53 response and apoptosis, but it can also lead to hematologic defects (2, 36, 47). One of the major reported side effects of MDM2 inhibition across different cancers has been thrombocytopenia (2). Our PDX study showed that tumor volumes in mice treated with milademetan continue to be substantially lower as compared with those in vehicle-treated mice for several days after withdrawal of treatment (Figure 4, A and B). This could mean that it is possible to have longer drug-free intervals between multiple rounds of treatment, which could potentially reduce side effects of the drug. Moreover, because milademetan was highly potent as compared with Nutlin-3a in our studies, lower required doses may also translate to decreased side effects. Our data collectively support the potential use of milademetan for WT p53 MCC. Approximately 80% MCC are MCCP tumors, and most MCCP tumors have WT p53 (16, 18, 20, 23, 24, 26). Of note, between 3% and 24% of MCCN tumors have been reported to contain WT p53 (16, 18, 19, 26, 48). Therefore, MCCN tumors with WT p53 could also respond to treatment with MDM2 inhibitors and inclusion of patients with MCCN tumors in clinical trials could be considered. This also supports the idea that, along with classification of MCCP and MCCN status, it will be beneficial to further stratify patients with MCC based on their *TP53* status for inclusion into clinical trials.

## Methods

### Cell culture.

MCC MKL-1, WaGa, PeTa, and MS-1 cell lines have been previously described (28, 29). SJSA-1, JAR, and SW962 cell lines were obtained from ATCC. The established MCC cell lines as well as SJSA-1 and JAR cell lines were routinely cultured using RPMI medium (GIBCO) supplemented with 10% FBS, Glutamax (GIBCO), and penicillin and streptomycin (GIBCO). The SW962 cell line was routinely cultured using DMEM medium (CORNING) supplemented with 10% FBS, Glutamax, penicillin, and streptomycin. MCC PDCLs MCC-301 and MCC-336 were generated from PDX tumors expanded in mice and cultured using the NeuroCult NS-A Human Proliferation Kit (StemCell Technologies) supplemented with 20 ng/mL FGF (StemCell Technologies), 20 ng/mL EGF (Life Technologies), and 0.02% heparin (StemCell Technologies). All cell lines were routinely tested for contamination using a Mycoplasma PCR detection kit (LiliF). Accutase (StemCell Technologies) was used to obtain single-cell suspensions of MCC cells for cell counting.

### EGFP/p53DD MKL-1 generation.

Sequences for EGFP (Addgene 25899; a gift from David Root, Broad Institute of MIT and Harvard, Cambridge, Massachusetts, USA) and p53DD (aa 1–14, 303–390; Addgene 11128; a gift from Christopher Counter, Duke University Medical Center, Durham, North Carolina, USA) were cloned into dox-inducible expression vector pLIX_402 (Addgene 41394) (41, 49). Lentivirus was generated in HEK 293T cells using packaging plasmids VSV-G (Addgene 12259) and psPAX2 (Addgene 12260), gifts from Didier Trono (EPFL, Lausanne, Switzerland). MKL-1 cells were transduced with lentivirus containing dox-inducible EGFP or p53 DD, and a positive, polyclonal population was selected via treatment with puromycin (1 μg/mL). Dox-inducible expression of EGFP and p53 DD was confirmed via WB.

### AAVS1/nontargeting control/p53-KO CRISPR/Cas9 generation.

sgRNA guides targeting adeno-associated virus integration site 1 (AAVS1; 5′–3′, GTCCCCTCCACCCCACAGTG), a nontargeting control (SCR; 5′–3′, GAACCCCTGATTGTATCCGCA), or p53 (5′–3′, CCATTGTTCAATATCGTCCG) were cloned into lentiCRISPRv2 using the recommended protocol. The lentiCRISPR v2 was a gift from Feng Zhang (Broad Institute of MIT and Harvard) (Addgene 52961) (50). Lentivirus was generated in HEK 293T cells using packaging plasmids VSV-G and psPAX2. MKL-1 cells were individually transduced with lentivirus containing the sgRNA constructs, and a positive, polyclonal population was selected via treatment with puromycin (1 μg/mL). Single-cell clones were selected from each polyclonal population via dilution plating and grown separately. Successful KO of p53 was confirmed via WB on the single-cell cloned population.

### Drugs/chemicals.

Nutlin-3a was obtained from Selleckchem. Milademetan was provided by Rain Therapeutics. For in vitro assays, Nutlin-3a and milademetan were dissolved in 100% DMSO to obtain a stock concentration of 10 mM. For in vivo assays, milademetan was dissolved in 0.5% methylcellulose. Dox was obtained from Takara Bio and dissolved in DMSO to obtain a 2 mg/mL stock concentration.

### Cell viability assays.

One thousand MCC cells were plated into each well of a 96-well plate. Each sample was plated in triplicate, and 3 biological repeats were performed for each experiment. Cells were treated with DMSO (vehicle), Nutlin-3a, or milademetan at indicated concentrations for 3 days (milademetan) or 5 days (Nutlin-3a). If the assay was for a 5-day duration, cells were topped with medium-containing drug or vehicle on day 3. For experiments in which dox induction was necessary, cells were treated with 1 μg/mL dox for 24 hours prior to treatment with drug. Cell Titer Glo 2.0 (Promega) was used as per the manufacturer’s instructions, and the 96-well plate was read for luminescence measurement. Raw luminescence values were normalized to the DMSO control to obtain relative viability counts.

### WB analysis.

Cells were pelleted and washed once with PBS supplemented with protease and phosphatase inhibitors (EMD Millipore). Cell pellets were resuspended in EBC lysis buffer solution (50 mM Tris-HCl, 200 mM NaCl, 0.5%NP-40, and 0.5 mM EDTA) supplemented with protease inhibitors, phosphatase inhibitors, and 2-mercaptoethanol (Bio-Rad), followed by incubation on ice for 10 minutes. Lysates were then clarified by centrifugation at 16,000*g* for 20 minutes at 4°C. The supernatant was transferred to a different tube, and protein concentration was determined using the Bio-Rad Bradford assay. Lysates were normalized so that each sample had the same total protein content, followed by addition of 6× SDS-reducing sample-loading buffer (Boston BioProducts) and boiling of samples at 95°C for 10 minutes. An equal volume of normalized cell lysates was run on a 4%–20% gradient gel (Bio-Rad) followed by transfer to a 0.2 μM nitrocellulose membrane. The membrane was blocked using 5% milk in TBST, followed by incubation with primary (diluted in blocking buffer) and appropriate secondary antibodies (Bethyl Laboratories, diluted in 1% TBST). Immobilon Western Chemiluminescent HRP substrate (MilliporeSigma) was used, and WB signal was captured using the G-box imaging system (Syngene). Raw blots were processed using ImageJ (NIH) software.

For protein isolation from tumor samples, tumors were crushed to smaller pieces in liquid nitrogen using a mortar and pestle. Smaller bits of tumor were resuspended in RIPA lysis buffer (Boston Biochemicals) supplemented with protease and phosphatase inhibitors and homogenized using a tissue homogenizer. Lysates were placed on ice for 10 minutes, followed by clarification by centrifugation. The WB protocol followed was similar to that used for cells.

The following primary antibodies were used for WB analysis according to manufacturer’s recommendations: PARP (CST, 9542S), cleaved PARP (CST, 32563S), p53 DO-7 (CST, 48818S), p53 DO-1 (Santa Cruz Biotechnology, SC-126), p53 A1 (Santa Cruz Biotechnology, SC-393031), p21 (CST, 2946S), PUMA (CST, 4976S), MDM2 (CST, 86934S), MDM4 (Abcam, ab243859), ST (made in-house, Ab5) (28, 42, 51), Vinculin (MilliporeSigma, V9131), and TBP (CST 8515S).

### Xenograft tolerability studies.

Four female NSG mice were treated with 100 mg/kg milademetan once daily orally for 10 days. The animals were weighed daily, and their body weight was noted. The 100 mg/kg dose was the highest dose used in the efficacy study.

### Xenograft efficacy studies.

5 × 10^6^ MKL-1 cells with 50% Matrigel were subcutaneously implanted in the right flank of 7-week-old female NSG mice. Tumor volumes were recorded twice per week until tumor volumes reached 110–305 mm^3^ (mean, 187.9 mm^3^). Animals were randomly divided into 4 groups (*n =* 8–9/group). The 4 groups included mice that would receive 0.5% methylcellulose (vehicle) as treatment once a day for 30 days or 3 different oral doses of milademetan, including 25 mg/kg, 50 mg/kg, and 100 mg/kg. Tumor volumes and body weights were recorded twice weekly. The study was allowed to progress until tumors reached maximum permissible size or when the study endpoint of 30 days was reached. Animals were euthanized if the tumor volume exceeded 2000 mm^3^. Tumor samples were collected from euthanized mice in each group at 0.5, 2, 6, or 24 hours after the dose (*n =* 2/time point/group) at the endpoint or study termination on day 30. Tumor samples were flash frozen in liquid nitrogen stored at –80°C until analysis.

### PDX pilot studies.

PDX 33043, PDX 48396, and PDX 96712 were used for pilot studies. A single MCC tumor fragment was implanted into a single mouse for the pilot studies (1 × 1 study). Once tumors reached an average size of 150–200 mm^3^ (mean, 183 mm^3^), mice were treated daily with vehicle or a 100 mg/kg dose of milademetan for 21 to 28 days. Tumor growth in these mice was followed until the mice in the vehicle group reached the predetermined study endpoint of 2000 mm^3^. Of note, we did not have enough tumor fragments banked for PDX 96712 and, therefore, had to euthanize the vehicle mouse earlier in the study.

### PDX efficacy studies.

The PDX tumors were expanded in mice. These mice were known as expansion mice. Tumors were harvested fresh from an expansion mouse. Pieces were cut into approximately 2 × 2 mm^3^ pieces. A small cut was made into the flank of the efficacy mouse, a path was created just under the skin with blunt scissors, and the fragment was dipped in Matrigel and put into the space created. The skin was stapled, and topical analgesic was applied. The staples were removed within 7 days. The mice were enrolled into the study on a rolling basis depending on when the tumor volumes reached between 106 mm^3^ and 156.8 mm^3^ (mean, 124.7 mm^3^) and were randomly divided into 1 of the 3 groups. The groups included mice that would receive 0.5% methylcellulose (vehicle) as treatment once a day for 28 days or 2 different doses of milademetan, including 50 mg/kg and 100 mg/kg, orally. Tumor volumes and body weights were recorded twice weekly. If a mouse was observed to have more than 15% body weight loss compared with weight at day 1, it was given a drug holiday until body weight recovered. In the 100 mg/kg dose, 1 animal (no. 555) received a drug holiday on day 22 but resumed dosing at day 23 (refer to Supplemental Table 6 for raw body weight values).

For blood draws, a peripheral cheek bleed into an EDTA tube was performed. For plasma separation, the tubes were placed on a rolling rocker table for a few minutes to mix in the EDTA and prevent clotting. The tubes were then centrifuged at 1500*g* for 10 minutes, and the plasma separated and collected into a labeled Eppendorf tube using a sterile pipette and stored in a –80°C freezer until processing.

### Human GDF-15 detection.

The human GDF-15 Quantikine ELISA kit (DGD150, Bio-Techne) was used as per the manufacturer’s instructions. The GDF-15 values were deduced by comparing sample absorbance values to a standard curve generated using recombinant GDF-15 provided in the kit.

### Statistics.

All curve fitting, data plotting, and statistical analysis were performed using GraphPad Prism version 9.3.1. The specific statistical tests used for each experiment include the Kaplan-Meier test, Bonferroni’s multiple-comparison test with 2-way ANOVA, and the log-rank Mantel-Cox test. *P* values of less than 0.05 were considered significant.

### Study approval.

All animal studies carried out were approved by the Dana-Farber Cancer Institute Institutional Animal Care and Use Committee. Relevant ethical regulations for animal research were followed while conducting the animal studies.

## Author contributions

VA, JAD, VGT, RCD, AK, KMS, and PCG designed the research studies. VA, AK, KMS, and BM conducted experiments and acquired the data. VA, JAD, AK, KMS, and PCG analyzed the data. TCF, VGT, and RCD generated and provided critical reagents. VA, JAD, KMS, AK, BM, TCF, PCG, VGT, and RCD wrote, edited, and provided critical review of the manuscript.

## Supplementary Material

Supplemental data

Supplemental tables 1-6

## Figures and Tables

**Table 1:**
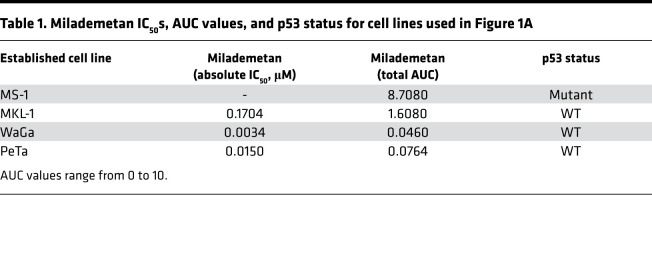
Milademetan IC_50_s, AUC values, and p53 status for cell lines used in Figure 1A

**Table 2:**
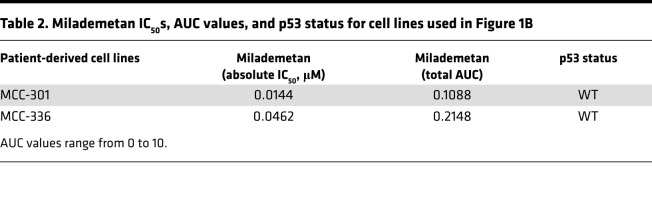
Milademetan IC_50_s, AUC values, and p53 status for cell lines used in Figure 1B

**Table 3:**
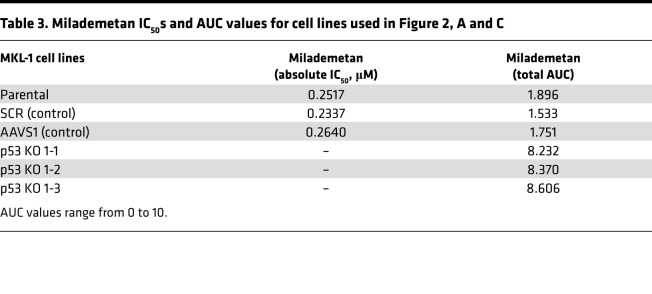
Milademetan IC_50_s and AUC values for cell lines used in Figure 2, A and C

**Table 4:**
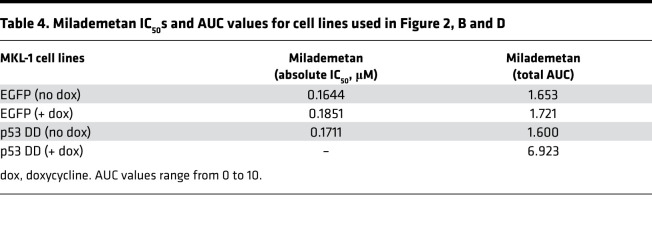
Milademetan IC_50_s and AUC values for cell lines used in Figure 2, B and D

**Table 5:**
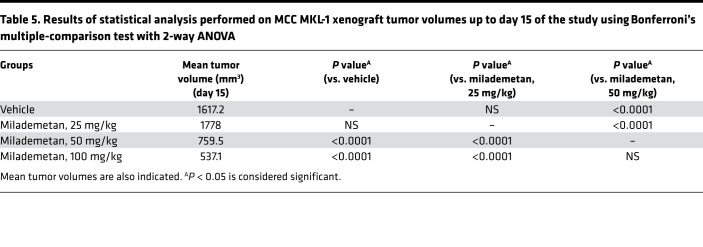
Results of statistical analysis performed on MCC MKL-1 xenograft tumor volumes up to day 15 of the study using Bonferroni’s multiple-comparison test with 2-way ANOVA

**Table 6:**
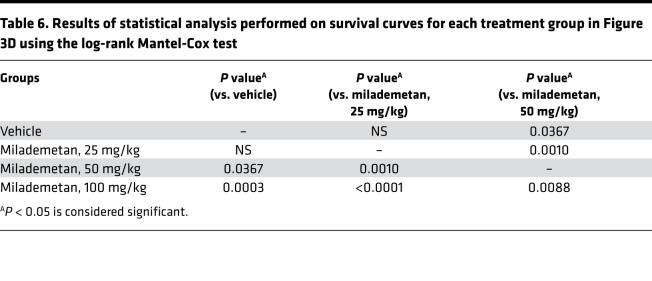
Results of statistical analysis performed on survival curves for each treatment group in Figure 3D using the log-rank Mantel-Cox test

**Table 7:**
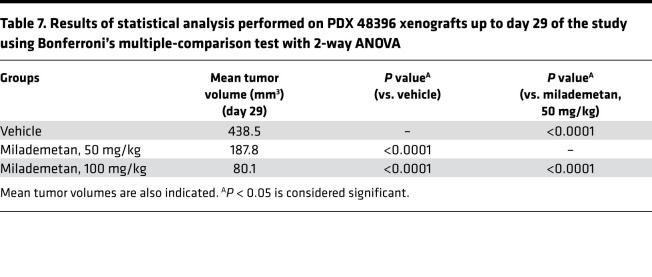
Results of statistical analysis performed on PDX 48396 xenografts up to day 29 of the study using Bonferroni’s multiple-comparison test with 2-way ANOVA

**Table 8:**
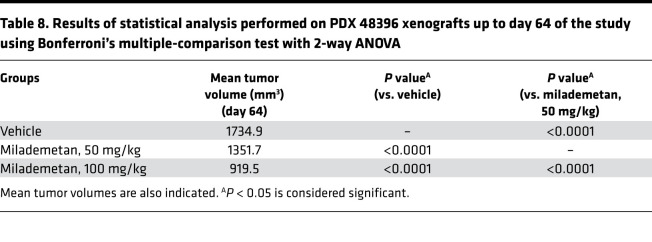
Results of statistical analysis performed on PDX 48396 xenografts up to day 64 of the study using Bonferroni’s multiple-comparison test with 2-way ANOVA

**Table 9:**
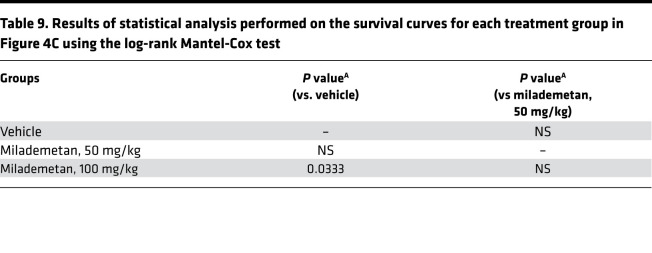
Results of statistical analysis performed on the survival curves for each treatment group in Figure 4C using the log-rank Mantel-Cox test

## Version 1 07/08/2022

Electronic publication
