# Supplementary material for: Milademetan is a highly potent MDM2 inhibitor in Merkel cell carcinoma
Source: JCI Insight. 2022 Jul 8;7(13):e160513. doi: 10.1172/jci.insight.160513 (PMC9310528; doi:10.1172/jci.insight.160513)

SUPPLEMENTAL FIGURES

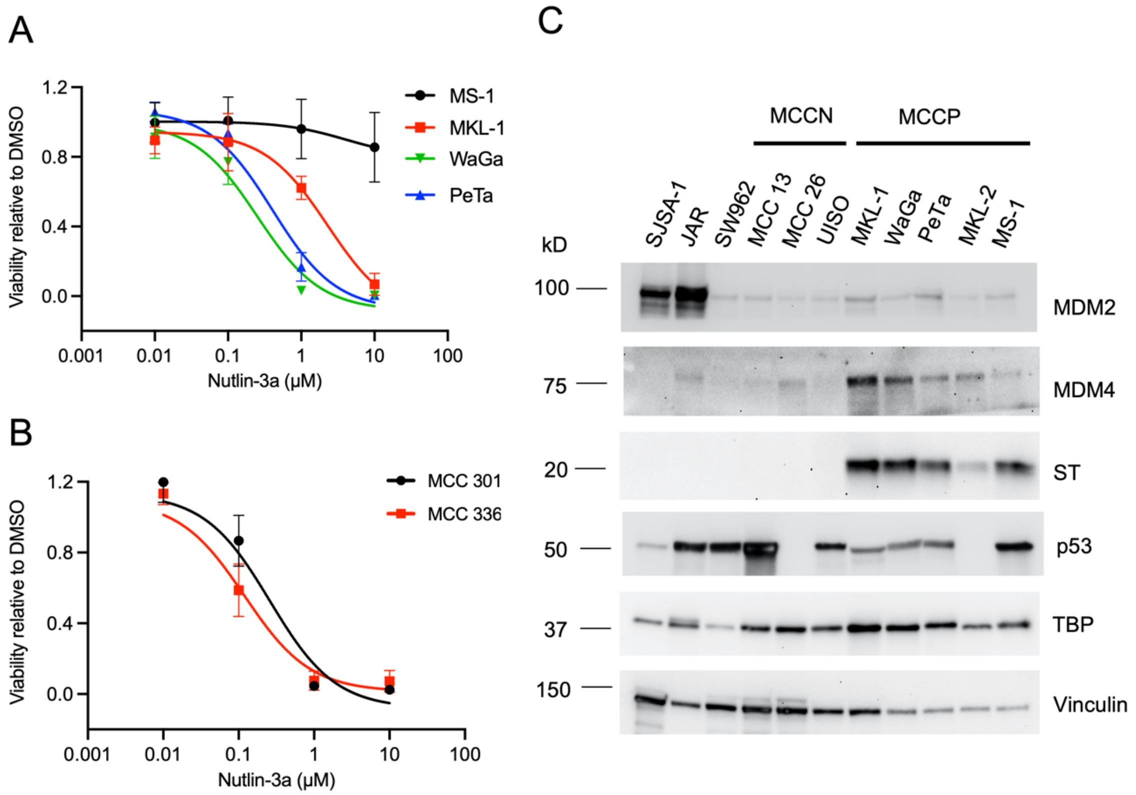

Supplemental Figure 1: MCCP cell lines with WT and functional p53 are sensitive to Nutlin-3a treatment.

(A and B) MKL-1, WaGa, PeTa and MS-1 cell lines or MCC PDCLs MCC 301 and MCC 336 were treated with indicated doses of Nutlin-3a and cell titer Glo assay was performed to assess effect on viability after five days of treatment. Each assay was performed in triplicate and three biological replicates were performed. Error bars indicate standard deviation. (C) WB indicates levels of MDM2, MDM4, MCPyV ST antigen and p53 across MCCN, MCCP cell lines. The panel also includes non-MCC SJSA-1, JAR and SW962 cell lines. TBP and Vinculin were used as loading controls. WB is representative of n=3.

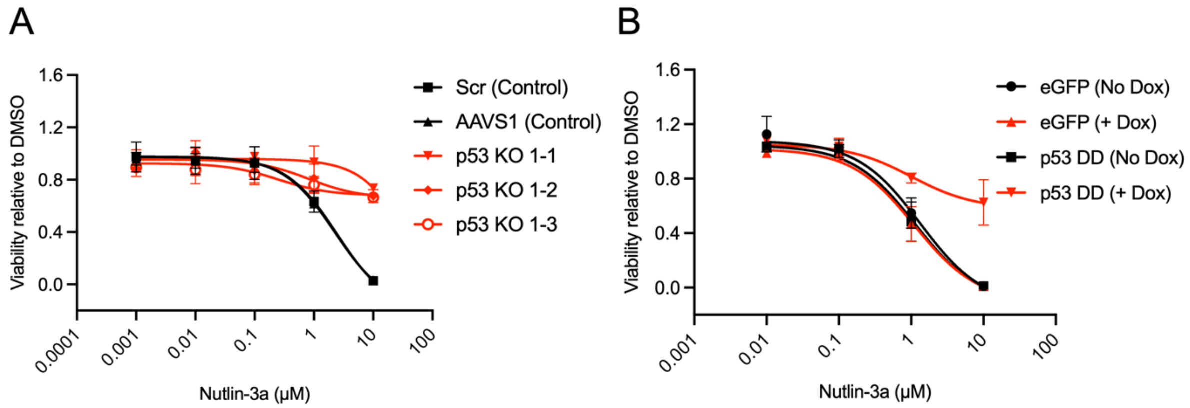

Supplemental Figure 2: **MCC cell lines devoid of p53 (p53 KO) or expressing dominant negative p53 (p53 DD) are resistant to Nutlin-3a treatment.**
(A and B) MKL-1 control or p53 KO cell lines in A and MKL-1 cell lines stably expressing GFP or p53 DD post 24 hour +/- Doxycycline (dox) induction in B were treated with Nutlin-3a followed by analysis of cell viability after 5 days. Each assay was performed in triplicates and three biological replicates were performed. Error bars indicate standard deviation.

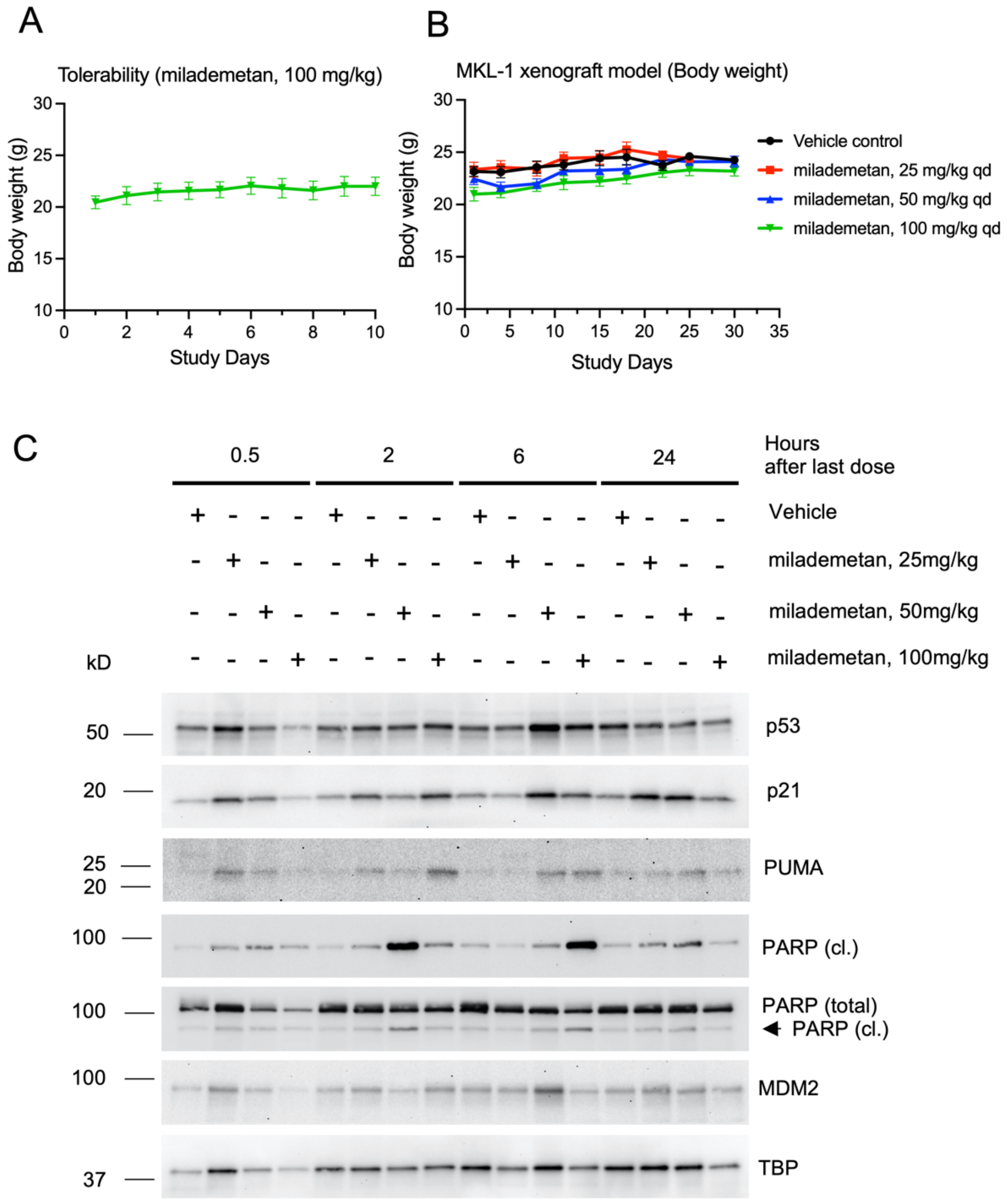

Supplemental Figure 3: Supplemental data for MCC MKL-1 Xenograft study

(A) Graph shows average body weight in grams of mice treated with 100 mg/kg of milademetan for a tolerability study. Error bars indicate standard error of mean. (B) Graph shows average body weight in grams of mice treated with either vehicle or indicated doses of milademetan throughout the duration of the MCC MKL-1 xenograft study. Error bars indicate standard error of mean. (C) WB analysis of 16 different tumors obtained from 16 individual mice at the indicated timepoints after the last dose of vehicle or milademetan was administered shows levels of indicated proteins.

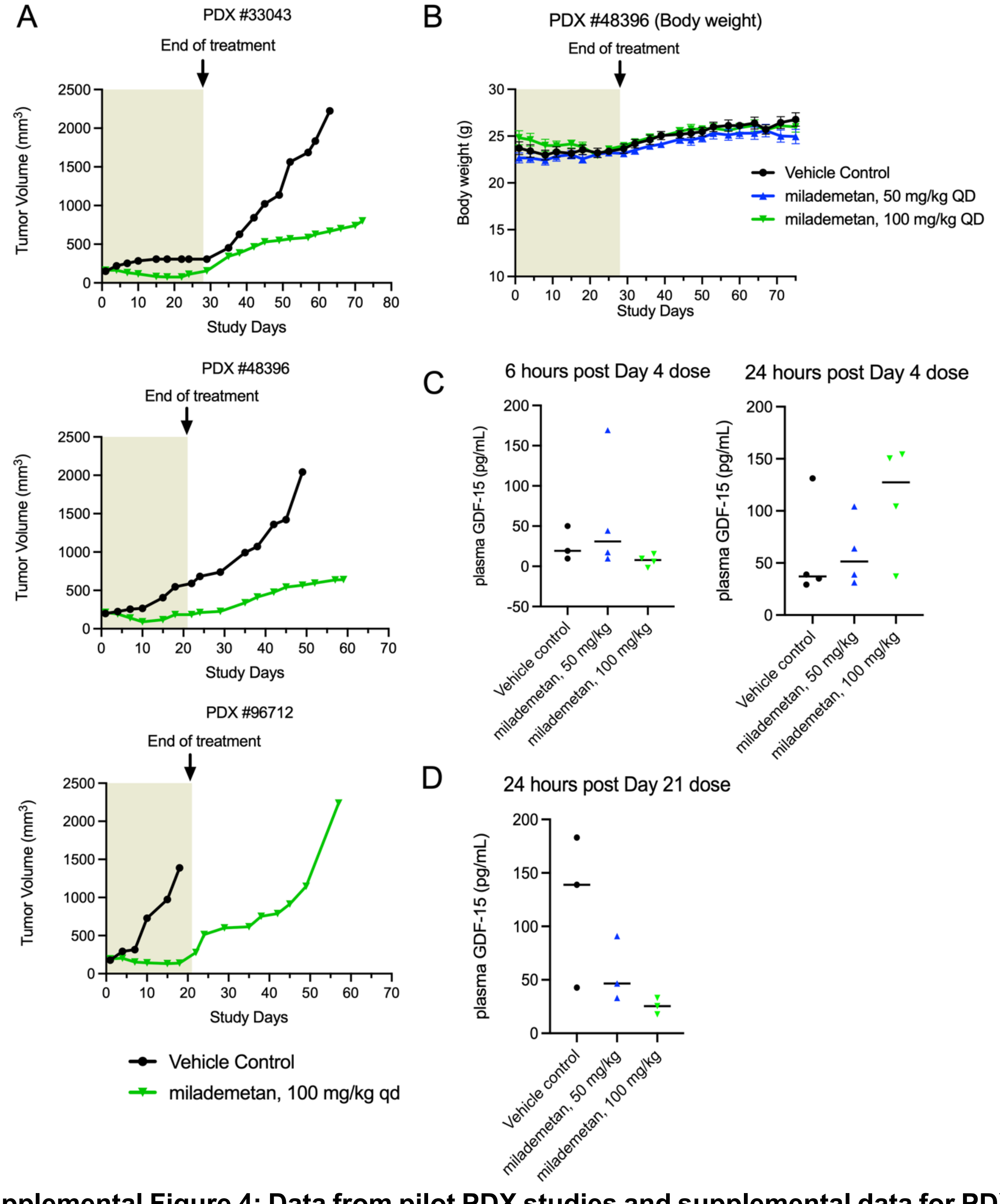

Supplemental Figure 4: Data from pilot PDX studies and supplemental data for PDX #48396 study.

(A) Graphs indicate tumor volumes in pilot studies for MCC PDX #33043, #48396 and #96712. A single tumor fragment was implanted in a single mouse for each condition. Tumor volumes of mice treated with vehicle or 100 mg/kg of milademetan during and post the course of treatment are shown. Shaded area indicates days when mice were treated with either vehicle or milademetan once a day. (B) Graph shows average body weight in grams of mice treated with either vehicle or indicated doses of milademetan throughout

the duration of the PDX #48396 study. Shaded area indicates days when mice were treated with either vehicle or the indicated milademetan dose once a day. Error bars indicate standard error of mean (C) Graph in C shows plasma GDF-15 levels measured from samples obtained at 6 hours (left) or 24 hours (right) post vehicle or milademetan on Day 4. (D) Graph in D shows plasma GDF-15 levels 24 hours post vehicle or milademetan dose on Day 21. No significant differences were observed. Each data point in C and D indicates GDF-15 levels detected in the plasma of an individual mouse.

SUPPLEMENTAL TABLES

Supplemental Table 1: Table denotes Nutlin-3a IC50's, AUC values and p53 status for cell lines used in Supplemental Figure 1A. AUC values range from 0 to 10.

Established cell line	Nutlin-3a (Absolute IC_{50}-µM)	Nutlin-3a (Total area under the curve)	p53 status
MS-1	-	9.158	Mutant
MKL-1	1.7060	3.8680	Wild-type
WaGa	0.2135	0.6091	Wild-type
PeTa	0.3513	1.3780	Wild-type

Supplemental Table 2: Table denotes Nutlin-3a IC50's, AUC values and p53 status for cell lines used in Supplemental Figure 1B. AUC values range from 0 to 10.

Patient derived cell line	Nutlin-3a (Absolute IC_{50}-μM)	Nutlin-3a (Total area under the curve)	p53 status
MCC-301	0.1571	2.6230	Wild-type
MCC-336	0.1030	2.3320	Wild-type

Supplemental Table 3: Nutlin-3a $IC_{50's}$ and AUC values for cell lines used in Supplemental Figure 2A. AUC values range from 0 to 10.

MKL-1 cell line	Nutlin-3a (Absolute IC_{50}-μM)	Nutlin-3a (Total area under the curve)
SCR (Control)	1.802	3.769
AAVS1 (Control)	1.442	3.733
p53 KO 1-1	-	8.475
p53 KO 1-2	-	7.538
p53 KO 1-3	-	7.219

Supplemental Table 4: Nutlin-3a $IC_{50's}$ and AUC values for cell lines used in Supplemental Figure 2B. AUC values range from 0 to 10.

MKL-1 cell line (+ Dox/ -Dox)	Nutlin-3a (Absolute IC_{50}-μM)	Nutlin-3a (Total area under the curve)
eGFP (No Dox)	1.044	3.327
eGFP (+ Dox)	0.938	2.912
p53 DD (No Dox)	0.956	3.002
p53 DD (+ Dox)	-	7.368

List of Tables included in Supplemental spreadsheet of large tables.

Supplemental Large Table 1: Absolute body weight (g) for animals in milademetan tolerability study.

Supplemental Large Table 2: Fate and absolute tumor volumes (mm^3) for animals in the MKL-1 xenograft study.

Supplemental Large Table 3: Fate and absolute body weight (g) for animals in MKL-1 xenograft study.

Supplemental Large Table 4: Absolute tumor volumes (mm^3) for animals in pilot PDX studies.

Supplemental Large Table 5: Fate and absolute tumor volumes (mm^3) for animals in PDX #48396 efficacy study.

Supplemental Large Table 6: Fate and absolute body weight (g) for animals in PDX #48396 efficacy study.

Full, uncut blots are provided as a separate supplemental file.

Full unedited blot
for Figure 1C

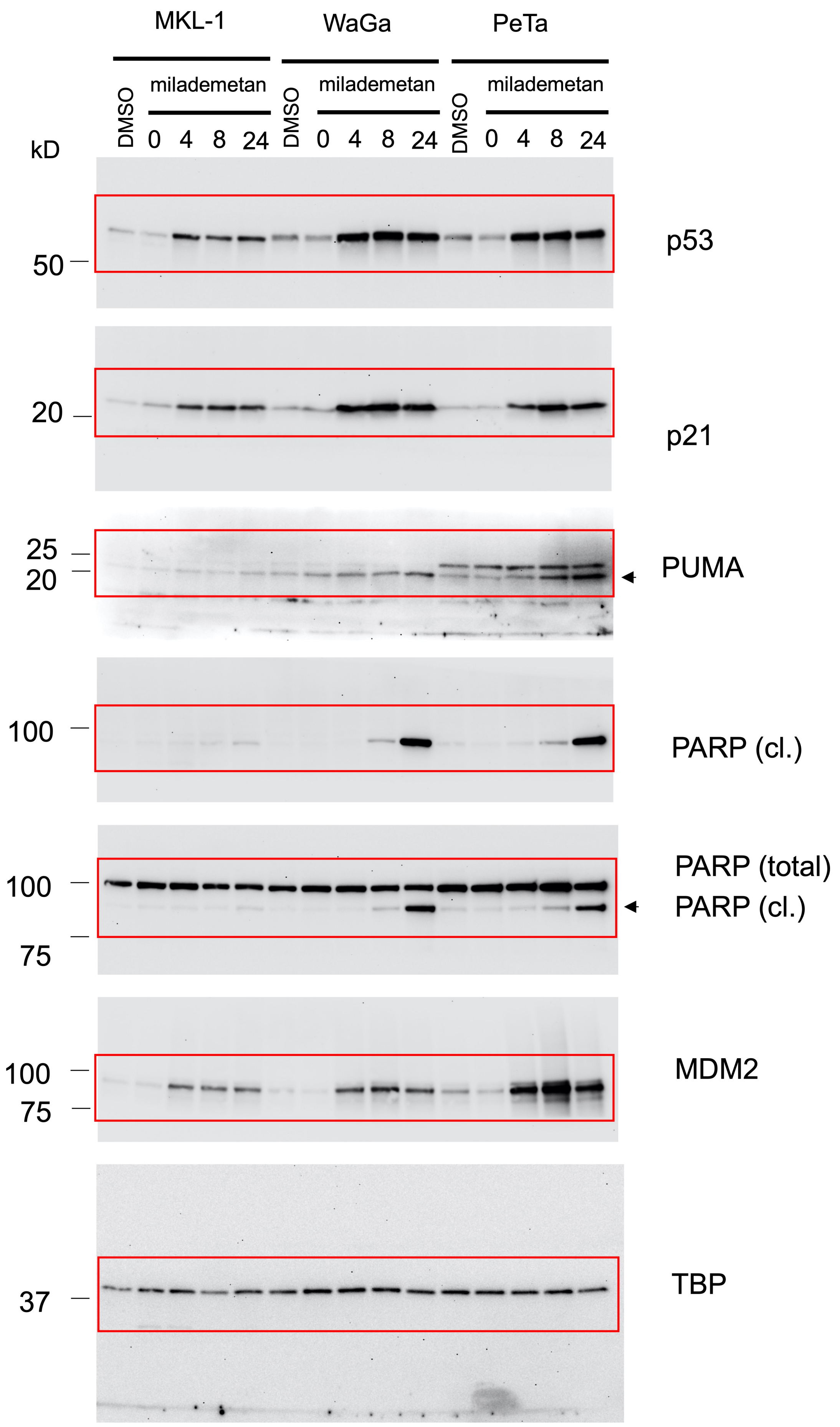

Full unedited blot for Figures 2A and 2B

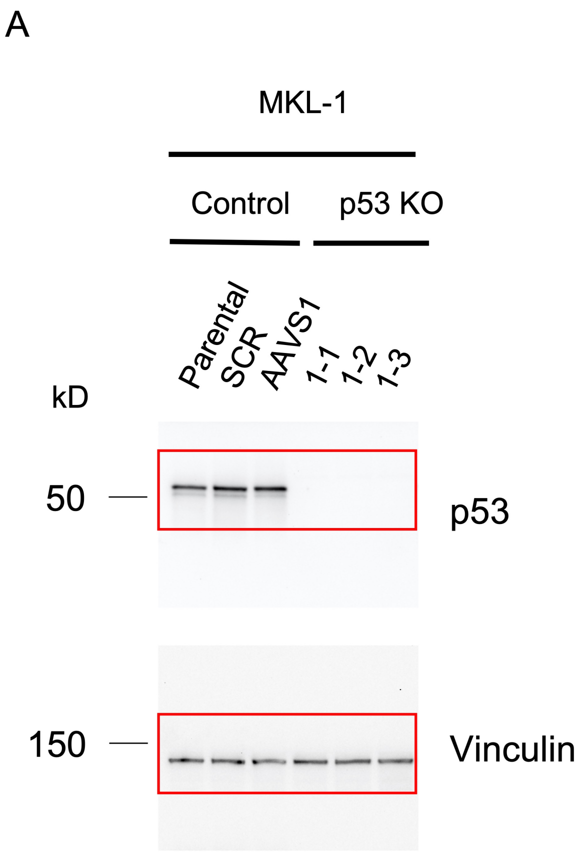

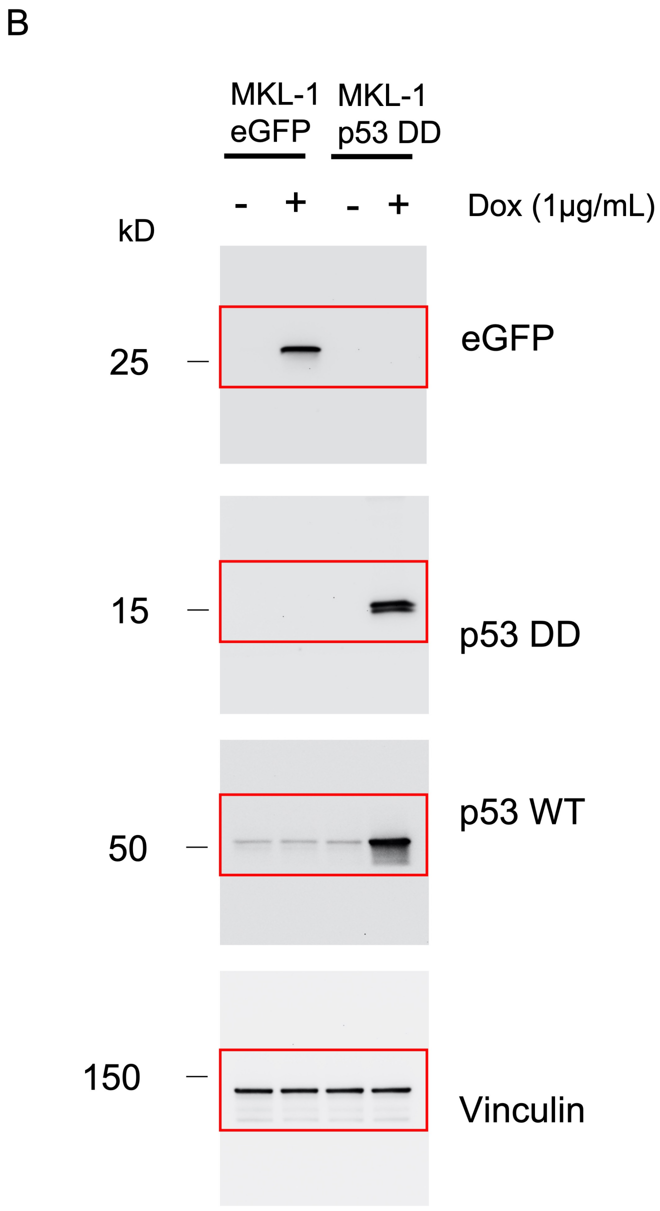

Full unedited gel for
Figure 3E

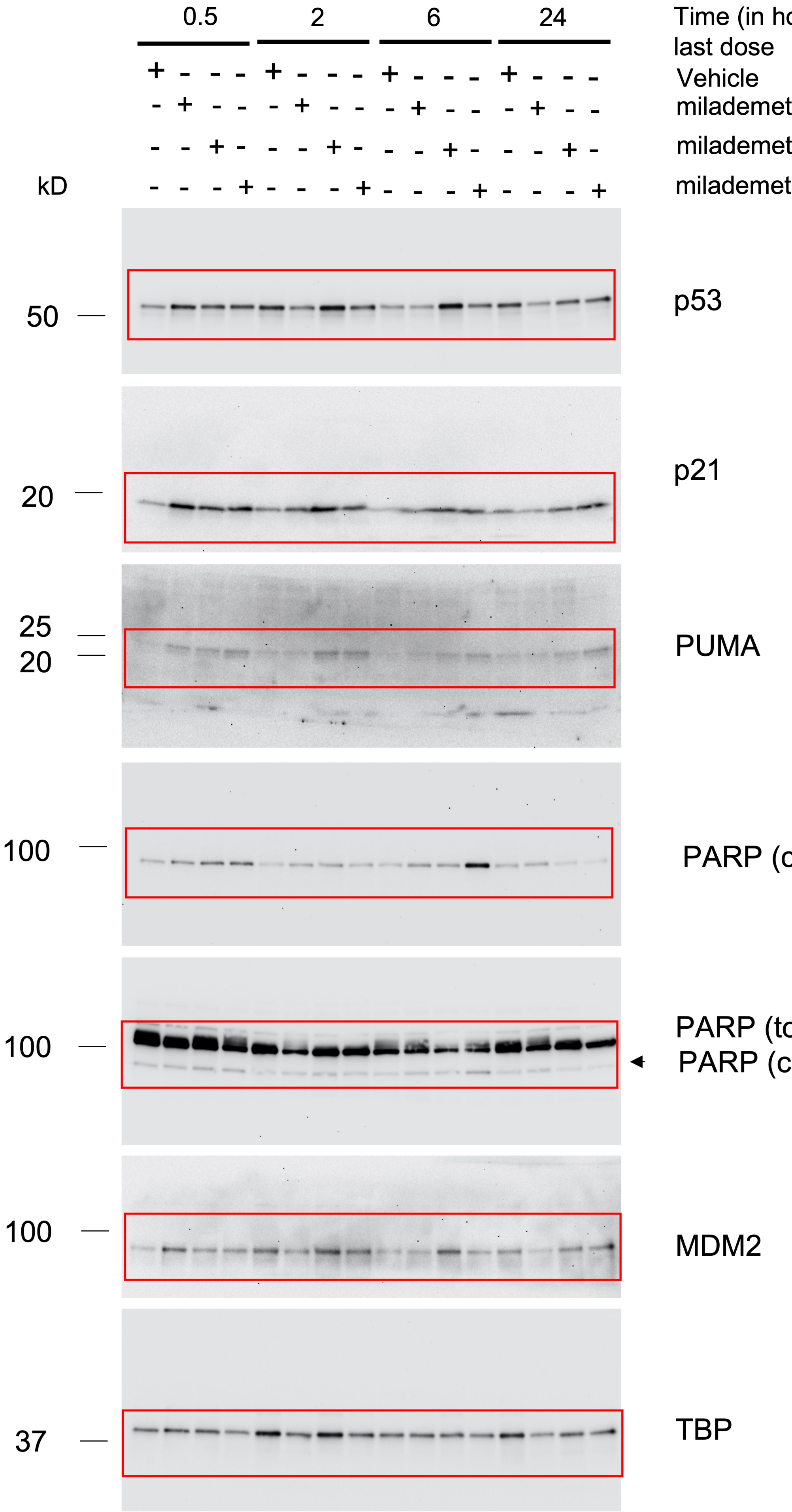

Full unedited blot for
Supplemental Figure 1C

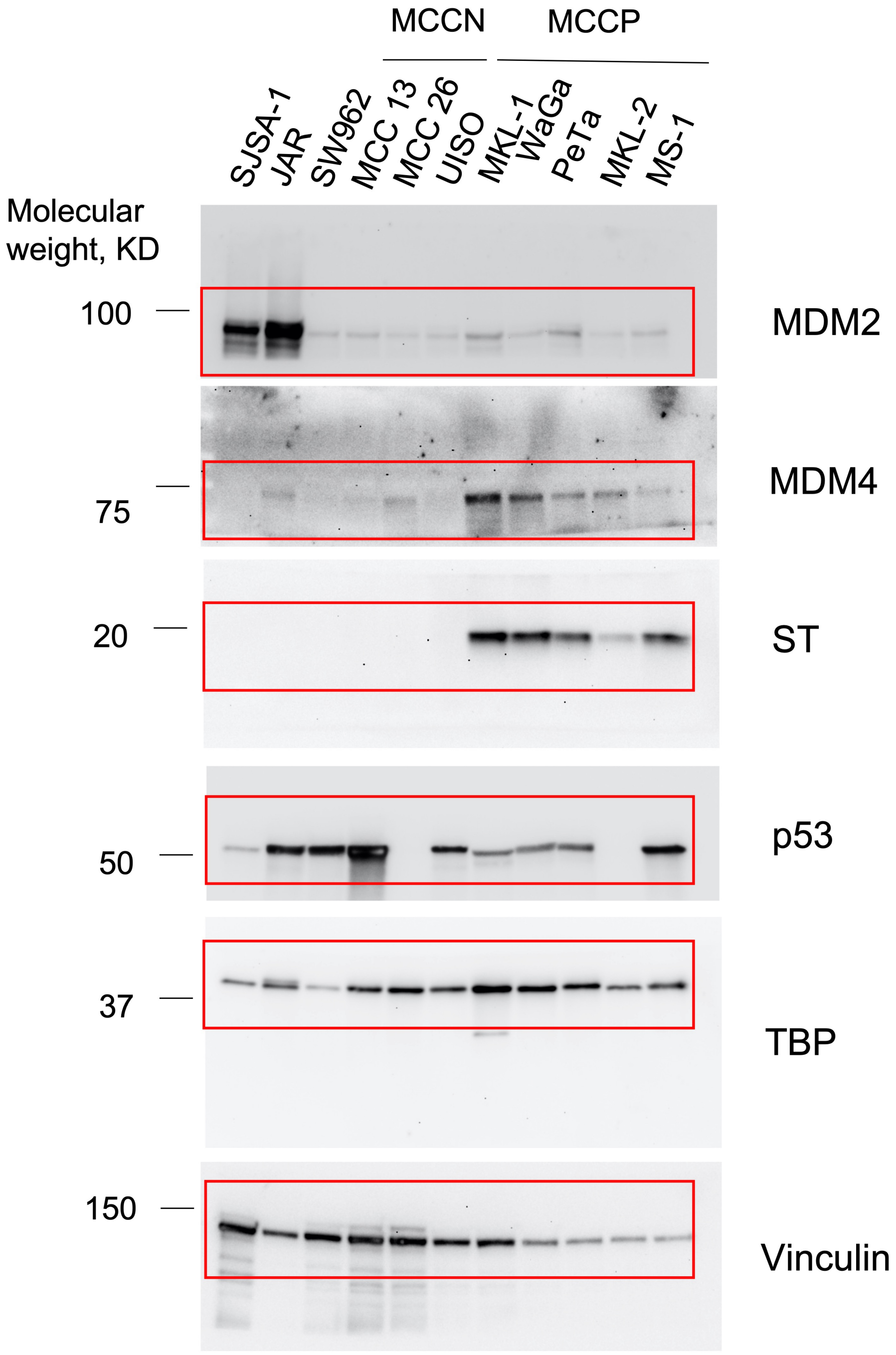

Full unedited blot for Supplemental Figure 3C

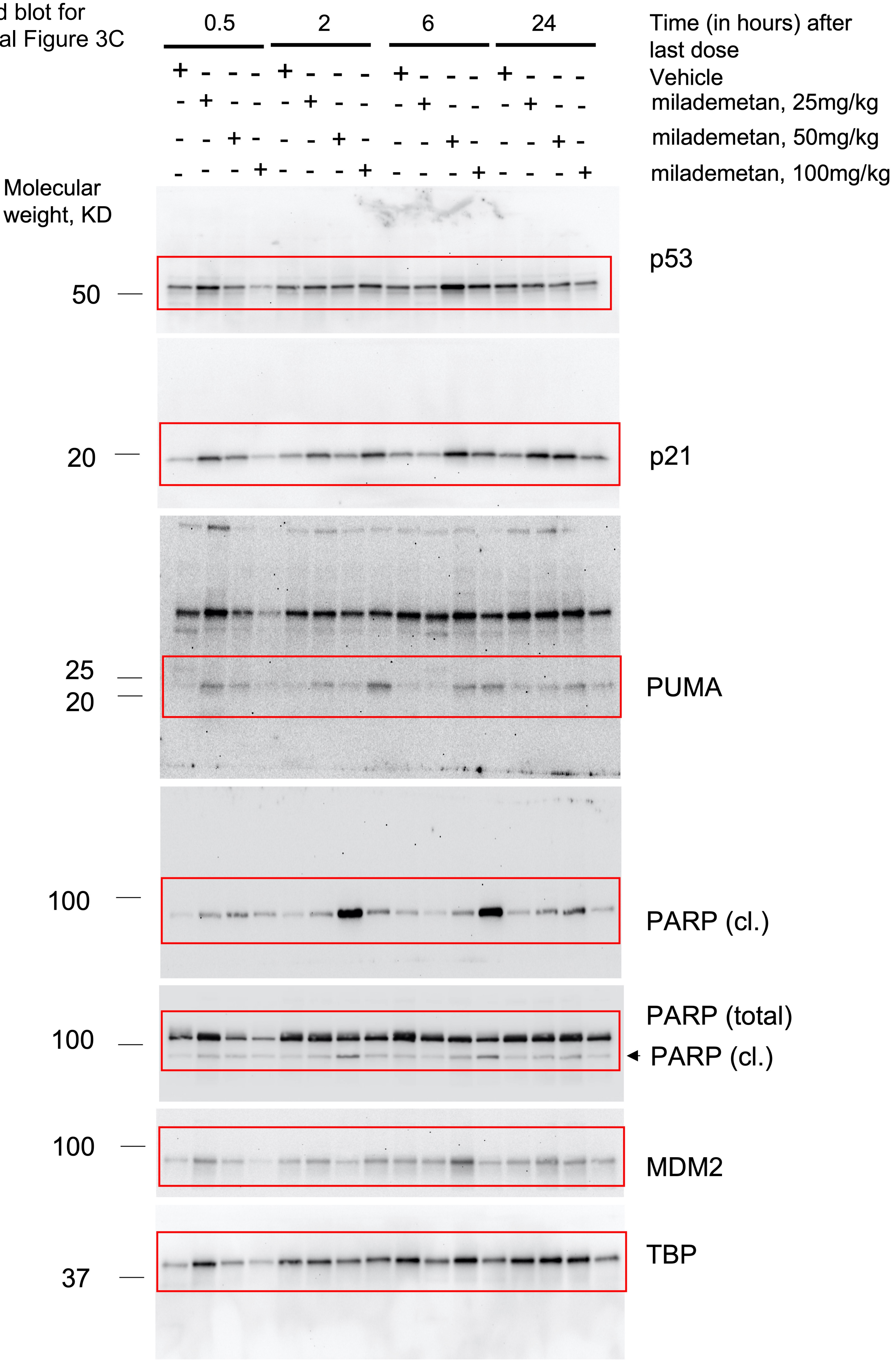

Supplement: Supplemental data [file jciinsight-7-160513-s213.pdf]
